# Effectiveness of peer-led education interventions on contraceptive use, unmet need, and demand among adolescent girls in Gedeo Zone, South Ethiopia. A cluster randomized controlled trial

**DOI:** 10.1080/16549716.2022.2160543

**Published:** 2023-01-25

**Authors:** Yohannes Addisu Wondimagegene, Gurmesa Tura Debelew, Zewdie Birhanu Koricha

**Affiliations:** a College of Health Sciences and Medicine, Department of Health, Behavior, and Society, Dilla University, Dilla, Ethiopia; b Institute of Health, Faculty of Public Health, Department of Population and Family Health, Jimma University, Jimma, Ethiopia; c Institute of Health, Faculty of Public Health, Department of Health, Behavior, and Society, Jimma University, Jimma, Ethiopia

**Keywords:** Peer-led education, contraceptive use, unmet need, contraceptive demand, student, high school

## Abstract

**Background:**

Peer-led education interventions are assumed to be an effective means of increasing contraceptive utilization and demand in adolescents. However evidence is lacking on whether peer-led education is effective in promoting the demand for and use of contraceptives in adolescent girls, especially in resource-limited settings.

**Objective:**

The present study evaluated the effectiveness of peer-led education interventions in improving contraceptive use, unmet needs, and demand among sexually active secondary school adolescent girls in Gedeo Zone, South Ethiopia.

**Methods:**

A single-blinded cluster randomised controlled trial study was performed in six randomly selected secondary schools in the Gedeo Zone, southern Ethiopia. A total of 224 participants were recruited and randomly assigned to the intervention and control groups. The intervention group received peer-led education intervention for six months. A pre-tested and validated questionnaire was used to measure contraceptive use, unmet need, and contraceptive demand. A generalised estimating equation (GEE) model was used to examine the effectiveness of the intervention.

**Result:**

After six months of intervention, the Differences-in-difference in contraceptive use, unmet need, and contraceptive demand between the intervention and control groups were 25.1%, 7.4%, and 17.7%, respectively. There was a statistically significant difference in contraceptive use [AOR = 8.7, 95% CI: (3.66, 20.83), unmet need for contraceptives [AOR = 6.2, 95% CI: (1.61, 24.36)] and contraceptive demand [AOR = 6.1, 95% CI: (2.43, 15.11)] between the intervention and control groups.

**Conclusions:**

School-based peer education intervention effectively improved contraceptive use and unmet needs in a low-resource setting and created demand in sexually active adolescent girls. These results support the potential utility of this approach in similar settings for the promotion of contraception use and demand.

## Background

Adolescence is the phase of life between the ages of 10 and 19 [1]. A significant number of adolescent worldwide are sexually active [2]. However, these adolescents are exposed to several reproductive health challenges and consequences due to limited access to and poor utilization of contraceptive services. For example, early pregnancy and childbirth in adolescents are the leading health problems in many low-income countries (LICs) [3]. Girls under 19 years of age have a 50% higher risk of stillbirth and neonatal death in addition to obstetric fistula, eclampsia, postpartum haemorrhage, and postpartum endometritis [2].

Adolescent girls in developing countries actively engage in unprotected sexual intercourse, and one-third do not use modern contraception [4]. These adolescents may experience unmet needs and demands for contraceptives because of their unique fertility preferences, availability and access to services, and behavioural and sociocultural factors [5,6]. The information available to adolescents may be given in a manner that is authoritarian, judgemental, or not adapted to the adolescents’ values and viewpoints [7].

Adolescents are frequently too embarrassed to talk about sexuality with their sexual partners, which makes them unable to articulate their reproductive desires [8]. Therefore, facility-based or provider-based sexuality education and promotion of contraceptive use would not sufficiently increase contraceptive use on their own [9].

This problem calls for the use of effective strategies that engage and empower adolescent girls for open discussions on safe sexual behaviours in general and contraceptive use in particular. Because most adolescent girls are in schools where they often spend a significant amount of their time with peers, a school-based peer education approach to promote contraceptive use is a potential strategy [10–12].

The Sustainable Development Goals (SDGs) agenda acknowledges the importance of reproductive health education at school [13]. Peers may influence adolescents contraceptive use [3], which suggests the importance of addressing friends or peers to create a supportive social environment for contraceptive use [14]. The present study evaluated the effectiveness of peer-led education interventions in improving contraceptive use, unmet needs, and demand in sexually active adolescent girls in the Gedeo Zone, south Ethiopia.

## Method and materials

### Setting and period

The study was performed in the Gedeo Zone of southern Ethiopia, 362 km from Addis Ababa, the capital of Ethiopia. The Zone is divided into nine districts: Bule, Gedeb, Kuchare, Wonago, Yirgachafe, Kedida Gubata, Raphe, and Chorso. There are three hospitals, 35 health centres, and 146 health posts in the Zone. The total population of the Zone is 1,193,162, and an estimated 351,983 are adolescents. There are 29 junior schools (26 public, two private, and one religious school) and nine secondary schools. A total of 34,445 students were enrolled in secondary school during the academic year [15]. The present study covered six secondary schools (public) located in the Zone. The study was performed from 10/09/2021-10/03/2022.

### Trial design

A single-blinded school-based parallel cluster-randomised controlled trial with a 1:1 allocation ratio was performed to evaluate the effectiveness of peer-led education interventions in generating contraceptive use, unmet needs, and demand in sexually active secondary school adolescent girls. This trial was registered under Clinical Trials.gov with identifier number PACTR 202,109,586,981,531.

### Participants

The study participants for this intervention were 224 sexually active secondary school adolescent girls aged 15 to 19 years who attended secondary school in the Gedeo Zone, southern Ethiopia. The study protocol is described per the SPIRIT (Standard Protocol Items Recommendations for Intervention Trials) checklist [16]. A cluster randomised controlled design was used to avoid contamination between the groups. Clusters were schools in the Gedeo Zone, southern Ethiopia.

### Inclusion and exclusion criteria

Governmental secondary schools in the Gedeo Zone, teachers of biology and chemistry, selected peer educators and randomly chosen sexually active students were included in this study. However, participants who refused to provide written and informed consent, who were seriously ill during the intervention, and who refused to continue the intervention were excluded from the study.

### Peer educator and teacher selection process

Four outstanding, responsible, and communicative students were selected as peer educators in the intervention school in each section. Two biology and chemistry teachers per school were also recruited. The primary role of the peer educator was to facilitate the peer education session. The role of the teacher was to monitor whether peer education activity was going according to schedule.

### The training of peer educators

Peer educators received a standard training program. First, peer educators and teachers attended a training session for 10 days. The training content included intensive teaching, group discussions, and knowledge competitions. The training theme was divided into four sections: general introduction to reproductive health, knowledge and skills required to be a peer educator, knowledge of modern contraceptive methods, and practical demonstration needed to be a peer educator. The external team provided extensive training to all trainers, including training exercises outside the study area. Before delivering peer education sessions, trainers passed written and oral exams. The training and field exercises took approximately 20 days to complete before trainers were allowed to teach classes as peer educators.

### Intervention procedure

Before the beginning of the intervention, letters of invitation were given to selected female adolescents aged 18–19 years and sent to parents/guardians of adolescents aged 15–17 attending six secondary schools. Peer educators disseminated information on modern contraception to their peers via exciting structures, such as school clubs [17]. Following the peer education training course, peer educators applied the knowledge and skills acquired in the training course to perform intervention activities.

A booklet, flipchart, and poster aided the peer education intervention. The intervention was delivered once weekly for one hour and lasted at least six months. The content focused on a general introduction to female and male sex organs, development of sexual negotiation skills, and knowledge about pregnancy, contraception, and the consequences of unwanted pregnancy. Teachers were not present in the classroom during the actual intervention activities. The teacher’s primary role was to monitor the overall activity of the peer education and verify that the intervention was delivered according to the protocol.

Peer educators took on a less formal part and approach and relied on participatory classroom teaching techniques to facilitate discussions, which involved poems, question-and-answer competitions, drama, and role play. An attendance sheet for each session was collected to determine who attended. Students must attend at least 80% of the sessions as a compliance criterion. The intervention’s organisation, content, and delivery were standardised as much as possible across the experimental schools. The control group received a similar SRH intervention after the study ended.

### Intervention fidelity

The investigators developed criteria to assess the intervention’s fidelity based on the standards for peer education programs guideline [18]. Checklists were used to determine the intervention design, content, peer educator training, peer education process, and intervention receipt. The content validity of the education material was ensured by sending two academic centre experts in reproductive health, health education and behavioural science. Two expert meetings with professionals from multiple disciplines were held to develop health learning materials.

To balance the differences, equal numbers of participants were drawn from eligible students for the intervention and control groups. To standardise the intervention within the intervention group, each student received an equal number of peer education sessions and contacts. Pre- and post-training tests and practical evaluations were used to assess peer educator knowledge and skill. Data entry clerks were blinded by labelling the groups with a non-identifiable unique number until the analysis was completed.

## Outcome measurements

### Primary outcomes

#### Contraceptive use

This was defined as the use of any one of the modern contraceptive methods during and after the intervention period (at the time of the end-line data survey).

#### Unmet need for contraceptives

This was defined as the proportion of sexually active respondents who did not want to become pregnant and intended to use contraceptives but failed to do so during and after the intervention was delivered. The minimum time frame for assessment was six months after the initiation of the intervention.

#### Contraceptive demand

This was defined as the proportion of sexually active respondents currently using contraception or participants exposed to an unmet need for contraceptives during and after the intervention period. The minimum time frame for assessment was six months after the initiation of the intervention.

### Sample size determination and sampling

The sample size was determined using G-Power statistical software version 3.1.5. The assumption for the sample size was that two population proportions have an estimated prevalence (P1) of modern contraceptive use among secondary school students of 57% [19]. An estimated increase of 10% post-intervention contraceptive use was assumed to be 67% (P2) with a 95% confidence interval, 80% power, and design effect of 2. By adding a 15% non-response rate, the final sample size was 1,698.

However, only 224 participants who were currently sexually active adolescents were included in the analysis. The study included 273 sections from six schools, with 81 sections from grade nine, 76 from grade 10, 64 from grade 11, and 52 from grade 12. Using identification numbers, the number of research participants in each school and grade level was determined and allocated proportionally based on the population size.

### Randomisation

After the first phase of baseline data collection, schools were stratified into individual assignments of intervention or control groups. From nine secondary schools (clusters) in the Gedeo Zone, six schools (clusters) were randomly selected for the study, which resulted in three intervention and three control groups.

**Figure 1.**
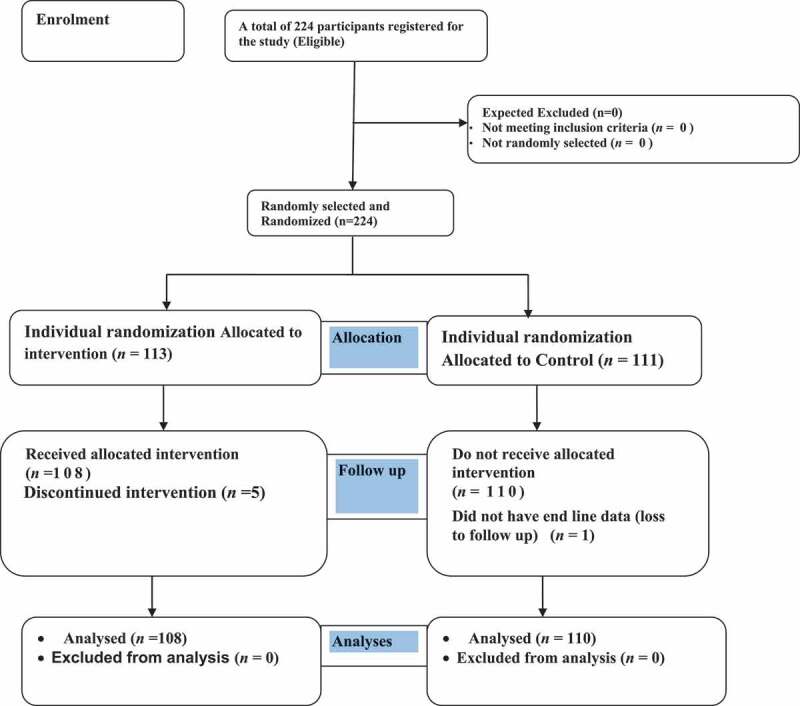
Consort flow diagram: Effectiveness of peer led education interventions in generating contraceptive utilization and demand among adolescents in Gedeo Zone, South Ethiopia.

The study arm associated with each randomly assigned number was written down prior to randomisation and placed in an envelope until all enrolled participants completed all baseline assessments and assigned interventions. Simple randomisation with a 1:1 allocation ratio was performed. The person responsible for randomisation notified the study arm for the intervention or control arm for the research team. Allocation concealment was not performed for study participants because the intervention was a training program, and it was impossible to blind schools or trainers to their study arm. The personnel responsible for subsequent data collection were blinded to group assignments. There was no change to the method after the trial began. Therefore, the study considered the consolidated standards of reporting trials (CONSORT) as a guide for study randomisation (Figure 1).

### Data collection procedure and tool

The study used a questionnaire adapted from the Ethiopia Demographic and Health Survey (EDHS 2014) and similar studies [20,21]. Before collecting the data, the tool was pre-tested in a similar setting. Twelve students and three midwives participated in the data collection process. The interview was performed at a private place in the school to ensure privacy.

### Data quality control

An experienced language expert translated the questionnaire from English into the local language and back-translated it into English to ensure consistency. A pre-test was performed to ensure the understandability and clarity of the questionnaire. Data collectors and supervisors received two-day training on the study’s objectives and how to perform the interview. The completeness, inconsistency, and data quality of the completed questionnaire were checked daily. Cronbach’s alpha of 0.7 or greater was used to assess the instrument’s reliability.

### Data processing and analysis

Data were entered into Epi Info version 7.2 software and exported to SPSS version 23 for analysis. Descriptive summaries, such as frequency and proportion, are presented. The group (intervention vs. control) difference was investigated using the chi-squared test for categorical variables. Pre- and post-intervention differences in difference between the intervention and control groups were measured. The generalised estimating equation (GEE) with a binary logit function was used to compare differences in outcome change between the intervention and control groups. The GEE accommodates clustered data and the correlation of observations within subjects. During model fitting, the unstructured covariance matrix was considered, and the effects of possible confounding socio-demographic variables, time, treatment, and time-treatment interactions were analysed. The impact of the intervention was evaluated using time and treatment interaction. Odds ratios and 95% confidence intervals were computed. A *P* value of 0.05 was considered statistically significant.

## Results

The study enrolled 218 eligible participants. Six participants were excluded from the study, five from the intervention arm and one from the control arm, for different reasons. Overall, the mean age of the participants was 16.97 (±1.38 ±SD) years. Most study participants in the intervention arm (91, 84.3%) and control arm (95, 86.4%) were unmarried. There were no statistically significant differences in age, religion, education level, place of residence, or marital status between the intervention and control arms (P > 0.05).

### Baseline contraceptive use-related characteristics of adolescents in secondary schools in the Gedeo Zone

There was a statistically significant difference in contraceptive method use between the intervention and control groups prior to the intervention program (P < 0.05). However, there was no statistically significant difference between the two groups in unmet need for contraceptives and contraceptive demand (P > 0.05) (Table 1).

**Table 1.** Baseline contraceptive use, unmet need and contraceptive demand in secondary schools in the Gedeo Zone.

Variables	Intervention group (n1=108) n (%)	Control group (n2=110) n (%)	P
**Contraceptive use**			0.016
Yes	75(69.4)	59(53.6)	
No	33(30.6)	51(46.4)	
**Unmet need for Contraceptive use**			0.313
Yes	16(14.8)	22(20.0)	
No	92(85.2%)	88(80.0)	
**Contraceptive Demand**			0.055
Yes	91(84.3)	81(73.6)	
No	17(15.7)	29(26.4)	

### Differences between baseline and end-line contraceptive-related characteristics of adolescents in the secondary school of the Gedeo Zone

Based on the per-protocol analysis, the proportion of participants using contraception increased 26% in the intervention group. This difference was statistically significant (P < 001 a). At the end of the study, the proportion of contraceptive use in the control group increased slightly by 0.9%. However, it was not statistically significant (P = 0.892 a). The Differences-in-difference in contraceptive use between the intervention and control groups was 25.1%, which was statistically significant (P < 001 a). At the end of the study, the proportion of unmet needs in the intervention and control groups among participants decreased 12.9% (P = 0.001 b) and 5.5% (P = 0.373 a), respectively. The Differences-in-difference in unmet needs between the intervention and control groups was 7.4%, which was statistically significant (P < 001 b). The proportion of contraceptive demand among participants increased 13.1% in the intervention group, which was statistically significant (P < 003 a). The percentage of contraceptive demand in the control group decreased 4.6% (P = 0.456 a) at the end of the study. The Differences-in-difference in contraceptive demand between the intervention and control groups was 17.7%, which was statistically significant (P < 001 b) (Table 2).

**Table 2.** Differences between baseline and end-line contraceptive use, unmet need, and contraceptive demand and the Differences-in-difference between intervention and control groups in secondary schools in the Gedeo Zone.

	Intervention group (%)				Control group (%)					
Outcome Variable	Baseline	End line	Difference (EL − BL) (%)	P value within group	Baseline	End line	Difference (EL − BL) (%)	P value within group	Difference in difference (%)	P value between group
Contraceptive use %	69.4	95.4	26	0.001^a^	53.6	54.5	0.9	0.892^a^	25.1	0.001^a^
Unmet need %	14.8	1.9	−12.9	0.001^b^	20.0	14.5	−5.5	0.373^a^	−7.4	0.001^b^
Contraceptive Demand %	84.2	97.3	13.1	0.003^a^	73.6	69.0	−4.6	0.456^a^	17.7	0.001^b^

^a^Pearson chi-squared, ^b^Fisher’s exact test, significant at *P* value <0.05 for both tests.

### Generalised estimating equation (GEE) intervention results of secondary school students in the Gedeo Zone

After controlling for potential confounders in the GEE multivariate model, the odds of sexually active adolescents using contraception in the intervention group were 8.7 times more likely in the intervention group than the control group [AOR = 8.7, 95% CI: 3.66, 20.83]. Unmet needs were six times more likely in the control group than the intervention group [AOR = 6.2, 95% CI: 1.61, 24.36]. After six months of intervention, contraception demand among adolescents in the intervention group was six times more likely than the control group [AOR = 6.0, 95% CI: 2.43, 15.11] (Table 3).

**Table 3.** GEE results of the intervention effect in secondary schools in the Gedeo Zone.

Variable		Beta coefficient	Standard error	AOR	95% CI	P
Contraceptive use	Intercept	0.14	0.19	1.15	0.79–1.68	0.44
	Time	0.67	0.28	1.96	1.12–3.42	0.01
	Group	0.03	0.08	1.03	0.88–1.21	0.65
	Time*group	2.16	0.44	8.73	3.66–20.83	0.01
Unmet need for Contraceptive	Intercept	1.38	0.23	4.00	2.50–6.38	0.01
	Time	0.36	0.36	1.43	0.70–2.91	0.31
	Group	0.38	0.15	1.46	1.08–1.98	0.01
	Time*group	1.83	0.69	6.27	1.61–24.36	0.01
Contraceptive Demand	Intercept	1.02	0.21	2.79	1.82–4.26	0.01
	Time	0.65	0.34	1.91	0.98–3.74	0.05
	Group	0-.22	0.09	0.80	0.66–0.96	0.02
	Time*group	1.80	0.46	6.06	2.43–15.11	0.01

## Discussion

There is a growing demand for sexual and reproductive health programs for adolescents in developing countries. However, there is not sufficient evidence on whether peer-led education effectively promotes the demand for and use of contraceptives among adolescent girls, especially in resource-limited settings. Therefore, rigorous contextual evaluations are needed to assess whether this behaviour is changed [22]. The current study was designed to promote the effectiveness of peer-led education intervention for contraceptive use, unmet need, and demand among sexually active secondary school adolescent girls in the Gedeo Zone. The intervention was designed based on findings from the baseline formative phase, which encompassed qualitative and quantitative studies. Therefore, it focused on a context-specific target population.

Different systematic review study findings showed that school-based peer-led intervention increased contraceptive use after five and 17 months of intervention [23] and led to a decrease in unwanted pregnancies [3]. Contraceptive use increased significantly after six months of school-based peer education intervention in the current study. This result suggests that school-based peer-led intervention decreases adverse reproductive health outcomes in students.

The application of peer education in out-of-school communities is also statistically significantly associated with better knowledge and use of modern contraceptives. Without a peer-education program, contraceptive use in the intervention community would have been significantly lower according to a studies from west Africa, Cameroon, rural Indonesia, and Spain [24–27]. However, studies from south Africa and India suggest that governments who advocate the widespread use of peer education as an approach to improve SRH of adolescents, including contraceptive use, need to recognise barriers to implementation and ensure on going monitoring and evaluation of effectiveness and cost-effectiveness [28,29]. This result suggests that testing the intervention in a specific context is critical before implementing large-scale peer education interventions on contraceptive utilisation that may demand high resources. School-based peer education intervention positively affected contraceptive knowledge, normative beliefs, and the attitude of adolescents in studies from India and Zambia [29,30].

Many quasi-experimental studies that included female and male adolescents from West Africa, south western Nigeria, and Cameron showed a positive effect after peer education intervention on the willingness to buy contraceptives, knowledge of contraceptives, attitudes toward contraceptives, and self-efficacy in contraception [24,31,32]. These results show that peer education has a positive effect on both sexes in influencing contraceptive use behaviour.

However, other studies indicated that educational intervention alone may not increase contraceptive use among adolescents. An intervention focusing on reproductive health services, including comprehensive post-abortion family planning services and financial incentives, produced positive changes in contraceptive utilisation and demand [3,4].

According to a review from low- and middle-income countries, the proportion of unmet needs for contraceptive use was reduced in an intervention group compared to a control group [33]. Unmet needs for contraceptive use decreased significantly after six months of school-based peer education intervention in the current study. The reduction in unmet needs after intervention suggested that adolescents who desired to use contraceptives before the intervention but failed to use contraceptives initiated use after peer-led education intervention in the intervention group.

Similar to different systematic reviews, the difference in contraceptive demand between the intervention and control groups increased significantly after peer education intervention in the current study [33,34]. This result shows that establishing and expanding peer education interventions helps students better understand contraceptive methods and significantly improves awareness of and demand for contraceptives. Adolescents are mostly exposed to sexual abuse but do not have a chance to demand contraceptives. Adolescents are mostly exposed to sexual abuse but do not have the circumstance to use contraceptives. However these patterns changed with peer-led education.

### Intervention

There is considerable resistance to providing contraceptive information to unmarried adolescents in many societies, which is embedded in community norms, sociocultural expectations, and contradictions prohibiting contraceptive use [35,36]. It is challenging to teach about contraception via community channels, such as community dialogue [37]. Schools are one of the best settings to initiate behaviour change because it is relatively free from religious leaders, community members, and parental pressure [21,38,39]. Therefore, the current study promoted peer education intervention as the best alternative to address contraceptive utilisation and demand among sexually active adolescent girls in secondary school. The strength of this study was that a formative study using qualitative and quantitative methods was performed to gain deep insight into barriers to the use of contraceptives among school-going adolescents as input for designing an intervention. The study used a cluster randomised controlled trial, which was the best design for understanding the intervention’s effectiveness. Findings from this study must be considered in light of their limitations. All outcomes reported in this review were based on self-report, which creates the potential for social desirability and recall bias. The study used a smaller sample because it did not include more clusters due to the limited number of public secondary schools in the study setting. The study excluded early-age (10–14) adolescent girls because most adolescents generally attend secondary school at age 15. Our study did not include male adolescents because they are less susceptible and less severely exposed to SRH problems. Therefore, they are not prioritised for the topic under study.

## Conclusion

School-based peer education intervention effectively improved contraceptive use and unmet needs in a low-resource setting and created demand among sexually active adolescent girls. This result supports the potential utility of the approach in similar settings for the promotion of contraception use and demand.

## Paper context

Pregnancy and childbirth among adolescents are major global health concerns. Adolescents in resource-poor settings have limited or no access to sexual and reproductive health education and contraception. School-aged adolescents are a significant target population for health promotion programs, particularly programs addressing sexual behaviours. School-based peer education intervention effectively improves contraceptive use, unmet needs, and demand among students. Similar programs should be implemented in areas where coverage of contraception use and demand is low.

## References

[1] Darroch JE, Woog V, Bankole A, Ashford LS. Adding it up: costs and benefits of meeting the contraceptive needs of adolescents. 2016.

[2] Salam RA, Faqqah A, Sajjad N, Lassi ZS, Das JK, Kaufman M, et al. Improving adolescent sexual and reproductive health: a systematic review of potential interventions. J Adolesc Health. 2016;59:S11–9. 27664592 10.1016/j.jadohealth.2016.05.022 PMC5026684

[3] Meherali S, Rehmani M, Ali S, Lassi ZS. Interventions and strategies to improve sexual and reproductive health outcomes among adolescents living in low-and middle-income countries: a systematic review and meta-analysis. Adolescents. 2021;1:363–390.

[4] Braeken D, Rondinelli IJIJoG, Obstetrics. Sexual and reproductive health needs of young people: matching needs with systems. Int J Gynaecol Obstet. 2012;119:S60–3. 22884824 10.1016/j.ijgo.2012.03.019

[5] MacQuarrie K. Unmet need for family planning among young women: levels and trends. ICF Int. 2014;12: 2016.

[6] de Vargas Nunes Coll C, Ewerling F, Hellwig F, De Barros AJD JRh. Contraception in adolescence: the influence of parity and marital status on contraceptive use in 73 low-and middle-income countries. Reprod Health. 2019;16:1–12. 30791914 10.1186/s12978-019-0686-9 PMC6383262

[7] McQueston K, Silverman R, Glassman AJSifp. The efficacy of interventions to reduce adolescent childbearing in low‐and middle‐income countries: a systematic review. Stud Fam Plann. 2013;44:369–388. 24323658 10.1111/j.1728-4465.2013.00365.x

[8] Presler-Marshall E, Jones N. Empowering girls to prevent early pregnancy. Oversees Dev Inst. 2012;1.

[9] Tsui AO, Brown W, Li Q. Contraceptive practice in sub-Saharan Africa. Popul Dev Rev. 2017;43:166. 29081552 10.1111/padr.12051 PMC5658050

[10] Birdthistle I, Vince-Whitman C. Reproductive health programs for young adults: school-based programs: pathfinder International, focus on young adults project watertown, MA, USA. 1997.

[11] Coyle K, Basen-Engquist K, Kirby D, Parcel G, Banspach S, Collins J, et al. Safer choices: reducing teen pregnancy, HIV, and STDs. Public Health Rep. 2001;116:82. 11889277 10.1093/phr/116.S1.82 PMC1913682

[12] Graham A, Moore L, Sharp D, Diamond I. Improving teenagers’ knowledge of emergency contraception: cluster randomised controlled trial of a teacher led intervention. BMJ. 2002;324:1179. 12016180 10.1136/bmj.324.7347.1179 PMC111106

[13] Joseph N, Mahato V, Pandey A, Mishra S, Prakash G, RJRH G. Experiences and perception towards reproductive health education among secondary school teachers in South India. Reprod Health. 2021;18:1–10. 34446038 10.1186/s12978-021-01224-6 PMC8394108

[14] Nsanya MK, Atchison CJ, Bottomley C, Doyle AM, Kapiga SH. Modern contraceptive use among sexually active women aged 15–19 years in North-Western Tanzania: results from the Adolescent 360 (A360) baseline survey. BMJ Open. 2019;9:e030485. 10.1136/bmjopen-2019-030485 PMC6720144 31467055

[15] Mola S, Aweke Z, Jemal B, Hussen R, Hailu S, Neme D, et al. Magnitude and associated factors for attitude and practice toward COVID-19 and its prevention among the residents of gedeo zone, southern ethiopia: a community-based cross-sectional study. Risk Manag Healthc Policy. 2021;14:253. 33519250 10.2147/RMHP.S277904 PMC7837529

[16] Moher D, Chan AW SPIRIT (standard protocol items: recommendations for interventional trials). Guidelines Reporting Health Research: a user’s manual. 2014:56–67.

[17] Glinski A, Sexton M, Petroni S. Understanding the adolescent family planning evidence base (review of literature). 2014.

[18] Fischer S, Fazekas Pederson K. Evidence based guidelines for youth peer education. NC: FHI Interagency Youth Working Group; 2010.

[19] Central Statistical Agency Addis Ababa, Ethiopia [Internet]. 2016.

[20] The DHS program ICF Rockville M, USA central statistical agency Addis Ababa. <EDHS-2016.pdf>. 2016.

[21] Melaku YA, Berhane Y, Kinsman J, Reda HL JBph. Sexual and reproductive health communication and awareness of contraceptive methods among secondary school female students, northern Ethiopia: a cross-sectional study. BMC Public Health. 2014;14:1–11. 24628909 10.1186/1471-2458-14-252 PMC4234022

[22] Hughes J, McCauley AP. Improving the fit: adolescents’ needs and future programs for sexual and reproductive health in developing countries. Stud Fam Plann. 1998;29:233–245. 9664634

[23] Lopez LM, Bernholc A, Chen M, Tolley EE. School‐based interventions for improving contraceptive use in adolescents. Cochrane Database Syst Rev. 2016. DOI:10.1002/14651858.CD012249 PMC9239532 27353385

[24] Brieger WR, Delano GE, Lane CG, Oladepo O, Oyediran KA. West African youth initiative: outcome of a reproductive health education program. J Adolesc Health. 2001;29:436–446. 11728893 10.1016/s1054-139x(01)00264-6

[25] Speizer IS, Tambashe BO, Tegang SP. An evaluation of the “Entre nous jeunes” peer—educator program for adolescents in Cameroon. Stud Fam Plann. 2001;32:339–351. 11831052 10.1111/j.1728-4465.2001.00339.x

[26] Nugroho E. Increasing modern contraceptive use among early marriage partners: an experimental study in rural Indonesia. Syst Rev Pharm. 2021;12:6.

[27] Diez E, Lopez MJ, Perez G, Garcia-Subirats I, Nebot L, Carreras R, et al. Impact of a community contraceptive counselling intervention on adolescent fertility rates: a quasi-experimental study. BMC Public Health. 2020;20:1–10. 31914967 10.1186/s12889-019-8122-1 PMC6950873

[28] Mason-Jones AJ, Mathews C, Flisher AJ. Can peer education make a difference? Evaluation of a South African adolescent peer education program to promote sexual and reproductive health. AIDS & Behav. 2011;15:1605–1611. 10.1007/s10461-011-0012-1 21809049

[29] Parwej S, Kumar R, Walia I, Aggarwal AK. Reproductive health education intervention trial. Indian J Pediatr. 2005;72:287–291. 15876753 10.1007/BF02724005

[30] Agha S, Van Rossem R. Impact of a school-based peer sexual health intervention on normative beliefs, risk perceptions, and sexual behavior of Zambian adolescents. J Adolesc Health. 2004;34:441–452. 15093801 10.1016/j.jadohealth.2003.07.016

[31] Ajuwon AJ, Brieger WR. Evaluation of a school-based reproductive health education program in rural South Western, Nigeria. Afr J Reprod Health. 2007;11:47–59. 20690287

[32] Agha S. A quasi-experimental study to assess the impact of four adolescent sexual health interventions in sub-Saharan Africa. Int Fam Plan Perspect. 2002;28:67–118.

[33] Denno DM, Hoopes AJ, Chandra-Mouli V. Effective strategies to provide adolescent sexual and reproductive health services and to increase demand and community support. J Adolesc Health. 2015;56:S22–41. 25528977 10.1016/j.jadohealth.2014.09.012

[34] Deitch J, Stark L. Adolescent demand for contraception and family planning services in low-and middle-income countries: a systematic review. Glob Public Health. 2019;14:1316–1334. 30794484 10.1080/17441692.2019.1583264

[35] Chandra-Mouli V, McCarraher DR, Phillips SJ, Williamson NE, Hainsworth G. Contraception for adolescents in low and middle income countries: needs, barriers, and access. Reprod Health. 2014;11:1–8. 24383405 10.1186/1742-4755-11-1 PMC3882494

[36] Abdul-Rahman L, Marrone G, Johansson A. Trends in contraceptive use among female adolescents in Ghana. Afr J Reprod Health. 2011;15:45–55. 22590892

[37] Nalwadda G, Mirembe F, Byamugisha J, Faxelid E JBph. Persistent high fertility in Uganda: young people recount obstacles and enabling factors to use of contraceptives. BMC Public Health. 2010;10:1–13. 20813069 10.1186/1471-2458-10-530 PMC2940919

[38] Hagan JE, Buxton CJJSR. Contraceptive knowledge, perceptions and use among adolescents in selected senior high schools in the central region of Ghana. J Sociol Res. 2012;3:170–180.

[39] Ngerageze I. Utilization of contraceptive methods among secondary school female adolescents at a selected secondary school in Rwamagana district. Rwanda: University of Rwanda; 2019. 10.4314/rjmhs.v5i1.9 PMC12240424 40641479

[40] GAotWm A. World medical association declaration of Helsinki: ethical principles for medical research involving human subjects. J Am Coll Dent. 2014;81:14–18. 25951678

